# The need for empathetic healthcare systems

**DOI:** 10.1136/medethics-2019-105921

**Published:** 2020-07-24

**Authors:** Angeliki Kerasidou, Kristine Bærøe, Zackary Berger, Amy E Caruso Brown

**Affiliations:** 1 The Ethox Centre and the Wellcome Centre for Ethics and Humanities, Nuffield Department of Population Health, University of Oxford, Oxford, UK; 2 Department of Global Public Health and Primary Care, University of Bergen, Bergen, Norway; 3 Berman Institute of Bioethics, Johns Hopkins University School of Medicine and Johns Hopkins Bloomberg School of Public Health, Baltimore, Maryland, USA; 4 Centre for Bioethics and Humanities, SUNY Upstate Medical University, Syracuse, New York, USA

**Keywords:** ethics, general

## Abstract

Medicine is not merely a job that requires technical expertise, but a profession concerned with making the best decisions and recommendations with reference to, and in consultation with, the patient. This means that the skill set required for healthcare professionals in order to provide good care is a combination of scientific knowledge, technical aptitude, and affective qualities or virtues such as compassion and empathy.

Being able to exercise empathy in healthcare depends on the individual healthcare practitioners and on the environment in which they work.^1 2^ It is, therefore, important to move away from an account of empathy that is only understood as a skill or virtue of the individual practitioner, and develop a new, broader account of healthcare-relevant empathy that encompasses healthcare systems and their role. In this paper, we develop and defend such an account by discussing: (1) Conditions for empathetic interaction between healthcare professionals and patients at the micro level. (2) Macro-level/meso-level governing policies that allow healthcare professionals to develop and exercise empathy, and patients to benefit from it.

## Introduction

Healthcare professionals are expected to have the right technical skills, medical knowledge and expertise, and to be empathetic, compassionate and trustworthy; and this is reflected in the training and education they receive. Yet numerous studies and reports demonstrate a decline in empathy among healthcare professionals.^3–5^

Exercising empathy in healthcare depends on the individual healthcare practitioners and on the environment in which they work.^1 2^ It is, therefore, important to develop a broader account of healthcare-relevant empathy that encompasses healthcare systems and their role, in addition to the skill or virtue of the individual practitioner. In this paper, we develop and defend such an account by discussing: (1) Conditions for empathetic interaction between healthcare professionals and patients at the micro level. (2) Macro-level/meso-level governing policies that allow healthcare professionals to develop and exercise empathy, and patients to benefit from it.

## Empathy as a professional skill and virtue

The medical profession does require technical expertise, and the ability to take the best decisions and recommendations with reference to, and in consultation with, the patient. This means that the skill set required for healthcare professionals in order to provide good care is a combination of scientific knowledge, technical aptitude, and affective qualities or virtues such as compassion and empathy.

It is frequently noted that paternalistic models of care fail to acknowledge the patient’s autonomy. The role of the healthcare professional in the patient-centred model is to present information to the patient, and to work with the patient to devise a plan of care that best fits her needs and values.^6^ In order for the nurse or physician to be able to propose appropriate care pathways, they need to engage with the patient and understand the situation from the patient’s perspective. While shared decision making has been held up as a corrective to paternalism, its account of the relationships between clinicians and patients remains thin. Knowledge of a patient’s values and preferences must be informed by an appreciation of their full personhood, and clinicians should also be able to express health-related values and preferences (and, one hopes, virtuous conduct) relevant to their professional identity. Thus, relational models of care have been proposed as a further corrective, acknowledging the partnership of patient and clinician based on mutual recognition of personhood.^6 7^

Qualities such as empathy and compassion are central in the relational model of care.^8^ Empathy is the ability to identify, understand and share another person’s feelings and perspective, while maintaining a self-other distinction.^1^ This means that the empathiser, although able to understand the other person’s situation and feelings, does not mistake their feelings as his or her own. The understanding of another person’s situation motivates feelings of compassion and the desire to help.^9^

Empathy is associated with a number of positive outcomes in healthcare, both for patients and healthcare professionals. For example, empathy can lead to greater patient satisfaction, trust and better adherence to treatment, as well as improved emotional health, symptom resolution, improved physiological measures and better pain control.^10 11^ Healthcare professionals also experience the benefits of empathy. Greater empathy can protect them from distress and burn-out and help them build better communication links with patients.^12 13^ Professional bodies list empathy and compassion among the skills and attitudes a healthcare professional should possess.^14^ Medical schools responsible for training the next generation of healthcare professionals screen students for empathy at the admissions stage.^15 16^ They also dedicate teaching time for the development of empathy and compassion as fundamental professional skills.^17^

Empathy is typically described as a cognitive and affective trait.^12 18^ Moral psychology and theory of mind experts have investigated ways in which empathy can be developed. Having experiences and interactions with a wider variety of individuals, finding oneself in different circumstances, and using imagination can help people understand situations from another perspective.^19^ Courses that seek to widen the social, moral and psychological experiences of students can help hone and foster skills of empathy and compassion.^20^ Yet, having good empathy skills is not enough for the expression of empathy. Expressing empathy also requires moral conviction, intention and orientation, akin to that required for the development and exercise of virtues. According to Aristotle, in order for a person to acquire virtues, she needs to want to do what is good (*kalon*) rather than what is not good, shameful or ugly (*aischron*). As Tong notes, people who engage only their cognitive capacities may be good at analysing how to achieve a certain goal, but are quite poor at determining whether achieving this goal is good or of value.^21^ The expression of empathy, therefore, as a professional skill and virtue requires the engagement of both cognitive and affective capacities as well as the intention and desire to do what is good. Teaching and learning, as well as habituation through lifelong reflection, action and relationships have been suggested as ways to promote empathy and compassion among healthcare professionals.^22^

## Barriers to empathy

Despite the value and importance of empathy and compassion in healthcare, in recent years there has been a notable decline of empathy among healthcare staff. Studies have followed the loss of empathy among medical students as they move through their years of training,^23^ empirical studies have demonstrated the low levels of empathetic engagement in clinical settings,^24 25^ and reports following care malpractices have highlighted a deficit of empathy in current medical practice.^4 26^ The development of courses teaching empathy, as mentioned above, has been a reaction to such acknowledgements of the decline of empathy in healthcare. Healthcare educators and institutions feel that they have to intensify their efforts to promote healthcare values such as empathy and compassion. Consequently, a number of new teaching techniques and evaluation methods have emerged to measure and monitor the effects of empathy teaching on the exercise of empathy on the ground, in clinical settings.^17 27–29^ Despite this increased attention on teaching empathy, studies still demonstrate that upholding these professional moral standards in practice is neither straightforward nor easily achieved.^25 30^

The reasons attributed to empathy decline in healthcare practice most often move beyond individual failings. It is not innate callousness or inability to engage with others that, in the majority of cases, leads to the absence of empathy and compassion in the therapeutic encounter. The factors most often cited include long working hours, understaffing, inability to spend sufficient time with patients, increased pressure to meet operational targets and increased workload.^1 25^ Professional bodies and institutions have responded to such findings by highlighting the value of empathy and compassion, and by coming up with plans to address the problem. For example, in the UK following the Francis Inquiry, which investigated failures of care at Mid-Staffordshire NHS Foundation Trust in 2013, the NHS created a new strategy intended to develop the culture of compassionate care, and outlined six areas of action, including measuring impact and embedding care values into effective training and recruitment strategies for staff.^31^

The majority of the interventions for the enhancement of empathy are often directed towards individuals. Whether through effective training and recruitment in medical schools or by helping existing staff develop skills to make ‘every contact (with patients) count’, the locus of attention has been the individual healthcare practitioner. This may be because, as mentioned above, empathy is perceived as an individual characteristic, or personal virtue that professionals are expected to demonstrate in their practice. When professionals fall short of this expectation, those in charge often look to the individual and try to identify reasons why that person might have failed to adhere to the established moral professional standards: Is it a knowledge deficit problem? Is it a personal moral shortcoming? Is it that the individual involved lacks resilience and cannot cope with the pressures of professional life? For each one of these problems, an answer is developed, such as mindfulness courses to help individual healthcare professionals cope with their high-pressure profession.^32 33^ Yet, focusing on the individual has meant that other factors that contribute to the decline of empathy in practice are often sidelined or ignored.

## Empathy and the role of systems

Studies have explored the impact of empathy training on the delivery of care, as well as how courses on resilience and mindfulness can help address the perceived deficit of empathy. However, there is one issue that, although acknowledged, is rarely explored. This is the role systems play in facilitating or impeding individuals’ efforts to behave in a way consistent with their professional values. A very broad approach to this issue is captured by the idea of a caring institution,^34^ and the suggestion that institutions ought to manifest empathetic concern for all people and ‘for those who promulgate, maintain, or participate in them’.^35^

The context and structure in which practitioners operate play an important role in their ability to act in certain ways, as well as contributing to their beliefs about how they ought to act. It is therefore important to ‘ensure that our institutions facilitate the formation and preservation of justified morally relevant beliefs (p. 110)’.^36^ One way that institutions can influence the moral beliefs and actions of individuals is by creating conditions that promote or incentivise certain practices and reinforce the holding of specific viewpoints. In the case of empathy and compassion in healthcare, a system that rewards processing patients quickly to meet operational targets, or rewards healthcare personnel for focusing on the illness rather than treating the patient holistically would reinforce the belief that skills such as empathetic engagement are not valuable or important and therefore ought not be prioritised. As O’Neil notes: ‘performance indicators have a deep effect on professional and institutional behaviour. […] If waiting lists can be reduced faster by concentrating on certain medical procedures, hospitals have reason to do so, even if medical priorities differ. *Perverse incentives are real incentives* (p. 55)’.^37^

Even if empathy is understood as a virtue rather than a skill, if the environment in which healthcare practitioners operate is not supportive of the expression of such a virtue, it is less likely that individuals operating in this environment will have the opportunity to develop it.^38^ It is through habituation that we acquire virtues, but without opportunity or space to practice such virtues, habituation is stifled and moral development hampered.

## Empathetic healthcare systems

Institutions and systemic structures play an important role in the expression, practice and habituation of empathy and compassion in healthcare. Although their role and influence has been acknowledged,^4^ the general tendency to regard empathy and compassion as merely professional skills or personal virtues has directed the focus on the individual rather than on systems and structures. We therefore propose that the notion of empathy is extended to include moral agents other than individuals; in this case systems and institutions. This way, attention can be directed to the ways in which systems and institutions can express and support empathy. It falls outside the remit of this paper to argue the reasonableness of considering collectives as distinct moral agents to which moral characteristics can be attributed; but this position has been defended elsewhere.^39^

We define empathetic systems or institutions as ‘systems and institutions that are structured and organised in such way as to create conditions that facilitate empathetic interactions in a non-arbitrary way throughout the whole service’. We distinguish between institutions and systems by referring to the former as distinct organised units of healthcare provision within a jurisdiction/nation, and the latter as the overall organisation of healthcare service within a jurisdiction/nation. Below, we present some preliminary thoughts on the organisational structures that could support the development and establishment of empathetic systems and institutions.

In order to investigate how healthcare systems can facilitate the practice of empathy on the ground, it is useful to distinguish among several levels of decision making, such as macro level, meso level and micro level.^40^ At the micro level, encounters between healthcare professionals and patients take place and clinical decisions are made. At meso level, decisions about the daily organisation of distinct healthcare institutions are made, while macro decisions concern the political organisation of institutions that make up a jurisdiction’s/nation's healthcare system. Macro decisions influence meso decisions, which in turn constrain conditions for clinical encounters and decision making at micro level. In this way, an empathic healthcare institution depends on a healthcare system governed by empathy-promoting political goals and decisions to facilitate empathic interpersonal interaction in a non-arbitrary manner.

Tronto argues for the importance of paying attention to the politics of care, in the form of power, purpose and particularity, as a way of building not just care institutions but caring ones.^34^ Recognising power in care-relationships and allowing political deliberation of interpretations of needs and the purpose of care, are also relevant elements for an analysis of how systems can impact on empathy. In general, how the purpose of the care and thereof derived needs for care are defined will impact on the conditions for assessing needs presented in the clinic including who is entitled to empathy and who is not. Furthermore, who is, within a decision-making hierarchy, delegated the authority to make these definitions can also influence the definitions and in turn the conditions. For example, if these definitions are made by politicians outside the institutions and without any deliberation and negotiation with healthcare personnel, they can end up dictating too narrow ideas of the purpose of care and a too limited set of interpretations of needs. Healthcare personnel can judge this unacceptable based on their caring-for relationships with patients and their hands-on experiences that might create a sense of solidarity and aversion of social injustice. A healthcare system that aims to provide adequate and empathic care, must therefore be self-reflexive regarding its own conditions of power. Moreover, it must cater for negotiation with the public, including those who are implementing the service, of what counts as needs and the purpose of care. Addressing power dimensions of care institutions emerges as a crucial self-regulating condition that in turn accommodates conditions for empathy.

### Structuring empathetic healthcare systems

Healthcare systems around the world are organised in different ways, reflecting sociopolitical, cultural and economic attitudes and beliefs. Some countries are treating healthcare as a basic good, basing access to healthcare on need rather than ability to pay, whereas others consider healthcare as a commodity to be subjected to market forces and rules, with many variations in between these two extremes. Different systemic structures could be characterised more or less empathetic depending on how they meet people’s care needs,^35^ and on how they invite those operating within them to consider and engage with each other. Healthcare systems that differentiate on patients based on income, occupation, race, gender, country of origin or immigration status, and so on, invariably create in-groups and out-groups, and thus appear more caring and empathetic towards some individuals rather than others. Also, by creating these different groups, they ‘other’ people and introduce boundaries between groups. We know that it is easier for one to be empathetic towards people she considers to be like her or part of her in-group, than people who are dissimilar to her or outside the group. Legislators concerned with developing fair healthcare systems should be cognisant of the effects healthcare system structures can have on the ability of the system to be empathetic and meet people’s care needs.^35^ They should try to design systems that encourage and acknowledge people’s commonality and interdependency, rather than systems that reinforce feelings of otherness, and boundaries between individuals and groups.^41^

### Creating conditions for empathetic interaction between healthcare professionals and patients

Deeper research is required in order to ascertain all the ways in which healthcare institutions and systems can promote or hinder the practice of empathy on the ground. However, current studies have already highlighted a number of factors which serve as barriers to empathy, including: increased operationalisation of care provision, increased focus on targets and protocols, understaffing, isolation of medical from social care, and systemic structures and practices that impede continuity of care.^4 25 30 42^ An empathetic healthcare system would need to actively reassess its priorities and structures in order to eliminate such adverse and limiting factors. Rethinking the indicators used to assess quality of care might be one way to achieve this.

In addition, it is important to acknowledge that institutions do not exist in isolation. Institutions might adopt rules and structures that faction as barriers to empathy not merely through their own incentives. Rather, institutional incentives are embedded in larger systems, often political systems, which determine the goals which themselves incentivise systems further downstream. To take one example, in the US healthcare system, reimbursement is provided for medications and procedures but not for interventions which address social determinants of health or emotional needs. It stands to reason that empathy is made more difficult when larger structures do not incentivise it, even if systems and institutions further downstream make such efforts.

A challenge, however, is that promoting empathy is not the only desired goal of macro-level, governing policies that are shaping institutions and framing the clinical encounter. Organising cost-effective and fair healthcare services, for example, are reasonable macro-level goals of healthcare systems that might appear to conflict with the aim of ensuring conditions for empathy. If such goals are implemented regardless of its impact on empathy, they can create tensions when introduced into institutions and clinical care between empathy and the other values they are set to promote (eg, cost-effectiveness). Institutional budget requirements can, for example, lead to understaffing that in turn constraint the conditions for empathy. Thus, an overall aim for an empathetic healthcare system would be to design a comprehensive set of macro-level policies that are sensitively constructed to avoid hampering healthcare workers' opportunities for developing the ability to exercising empathy at the micro level.

### Introducing policies that allow healthcare professionals to develop and exercise empathy

It is not reasonable for healthcare institutions and systems to require that their staff behaves empathetically, without paying attention to and creating structures and policies that permit the development and flourishing of empathy.^1^ Healthcare institutions need to demonstrate care and empathy towards the healthcare professionals serving under them. For example, they should help staff develop resilience in the face of the emotional labour of providing high quality empathetic care, tend to their psychological and emotional well-being, and also protect them from conditions of overwork, underpay and unfair treatment that leads to burn-out and feelings of being undervalued.

Also, attention towards empathy and the importance of achieving it on the micro level should be explicitly stated as a priority goal for healthcare systems and built into all governing instruments, including in measuring, assessing and evaluating strategies. In addition, providing empathic care should be an explicit aim of all healthcare professions. Macro-level decisions can also demand that healthcare personnel on the top of the hierarchical order are held responsible for ensuring and maintaining focus on empathy in the clinic and for facilitating easily accessible channels for the reporting of conditions that hamper empathy.

### Considering organisational structures that render the healthcare system empathetic

Our suggested definition of an empathic healthcare institution/system above emphasises the importance of systems and institutions to be structured and organised in such a way that they create conditions for empathetic interaction between healthcare professionals and patients *‘in a non-arbitrary way throughout the whole service’*. A system is not empathic if only one group of healthcare professionals works under conditions that allow them to foster empathy in the service they provide, if only some institutions are organised with empathy in mind, or just one phase of the care pathway leaves room for healthcare personnel to demonstrate empathy. The empathy of a system is reflected in the overall strategy of meeting all healthcare needs empathetically throughout the system.

In order to realise systems as comprehensively and consistently empathic, empathy must be intentionally facilitated as part of all areas of the system a user may encounter. A substantive way of achieving this might be to follow the Levesque *et al* user access framework to identify areas where empathy can be directly experienced by a patient.^43^ According to Levesque access to healthcare should be centred around five dimensions: (1) Approachability. 2) Acceptability. (3) Availability and accommodation. (4) Affordability. (5) Appropriateness. Following this framework an empathic healthcare system or institution would fulfil the follow conditions: First, such a system or institution must be accessible to anyone with a healthcare need, independently of other characteristics (eg, legal status). Second, the services provided must be acceptable to all, irrespective of cultural and social backgrounds. Third, services should be able to accommodate patients’ actual needs and living conditions. Fourth, they must be affordable, to avoid allowing arbitrary conditions, such as individual wealth, to determine care. Fifth and last, the provided service must be appropriate in terms of healthcare providers demonstrating empathy, such that patients are able to engage with their caregivers. All of these dimensions—in addition to care for the conditions of employees to develop and cultivate empathy—are required to entitle a healthcare system to the description *empathetic*.

While macro-level decisions taken by health authorities are subjected to the requirements suggested above, meso-level decisions carried out for distinct intuitions must be related to specific function of the institution within the comprehensively developed system. Yet, as underscored above, in order to foster empathy within institutions, there must be deliberation and negotiation over the politics and structure of care and healthcare institutions.

## Conclusion

Broadening the definition of empathetic healthcare to include healthcare institutions and systems requires a broader understanding of what makes empathy possible at macro, meso and micro levels. Rather than centring on the individual healthcare professional, a multitiered view is needed and should be the focus of future work on empathy in healthcare systems.

To realise an empathic healthcare system requires a much broader scope of macro-level decisions that explicitly and coherently relate to empathy than what has been discussed in the literature thus far. Seen in relation to Buchanan's social moral epistemology,^36^ the shaping of empathic healthcare systems or institutions on such extensive terms would be expected to strengthen positive attitudes towards empathy in everyone involved in developing and upholding these systems or institutions. Moreover, an empathic healthcare system or institution can now be positively and in more detail described on ideal terms as involving policy decision regarding targeted training of empathy in healthcare professionals, ensuring conditions for cultivating empathy amonth healthcare professionals, delegating responsiblity for overseeing empathy in clinical work, as well as intending developing implementation and evaluation of empathy promoting policies across all phases of heatlhcare access and provision.
